# Supplementary material for: Inactivation of the Kv2.1 channel through electromechanical coupling
Source: Nature. 2023 Sep 27;622(7982):410–7. doi: 10.1038/s41586-023-06582-8 (PMC10567553; doi:10.1038/s41586-023-06582-8)
Supplement: Supplementary file 1 — Supplementary Figs. 1–4 and Table 1. [file 41586_2023_6582_MOESM1_ESM.pdf]

nature portfolio

https://doi.org/10.1038/s41586-023-06582-8

Supplementary information

Inactivation of the Kv2.1 channel through electromechanical coupling

In the format provided by the authors and unedited

Supplementary information

Inactivation of the Kv2.1 channel through electromechanical coupling

Ana I. Fernández-Mariño [1 †], Xiao-Feng Tan [1 †], Chanhyung Bae [1 †], Kate Huffer [1,2],

Jiansen Jiang [3] and Kenton J. Swartz [1*]

[1] Molecular Physiology and Biophysics Section, Porter Neuroscience Research Center, National Institute of Neurological Disorders and Stroke, National Institutes of Health, Bethesda, MD 20892

[2] Department of Biology, Johns Hopkins University, 3400 N. Charles Street, Baltimore, MD 21218

[3] Laboratory of Membrane Proteins and Structural Biology and Biophysics Center, National Heart, Lung, and Blood Institute, National Institutes of Health, Bethesda, MD 20892

† These authors contributed equally to this work

* Corresponding author. Email: swartzk@ninds.nih.gov

Table of Contents

Page 3 Supplementary Information Figure 1|
Data processing workflow for the structure of Kv2.1 (1-598)

Page 4 Supplementary Information Figure 2|
Data processing workflow for the structure of the L403A mutant of Kv2.1 (1-598).

Page 5 Supplementary Information Table 1|

Pages 6-7 Supplementary Information Figure 3|
Structure-based sequence alignment of Kv, Cav and Nav channels

Page 8 Supplementary Information Figure 4|
Uncropped gel from Extended Data Fig. 1d

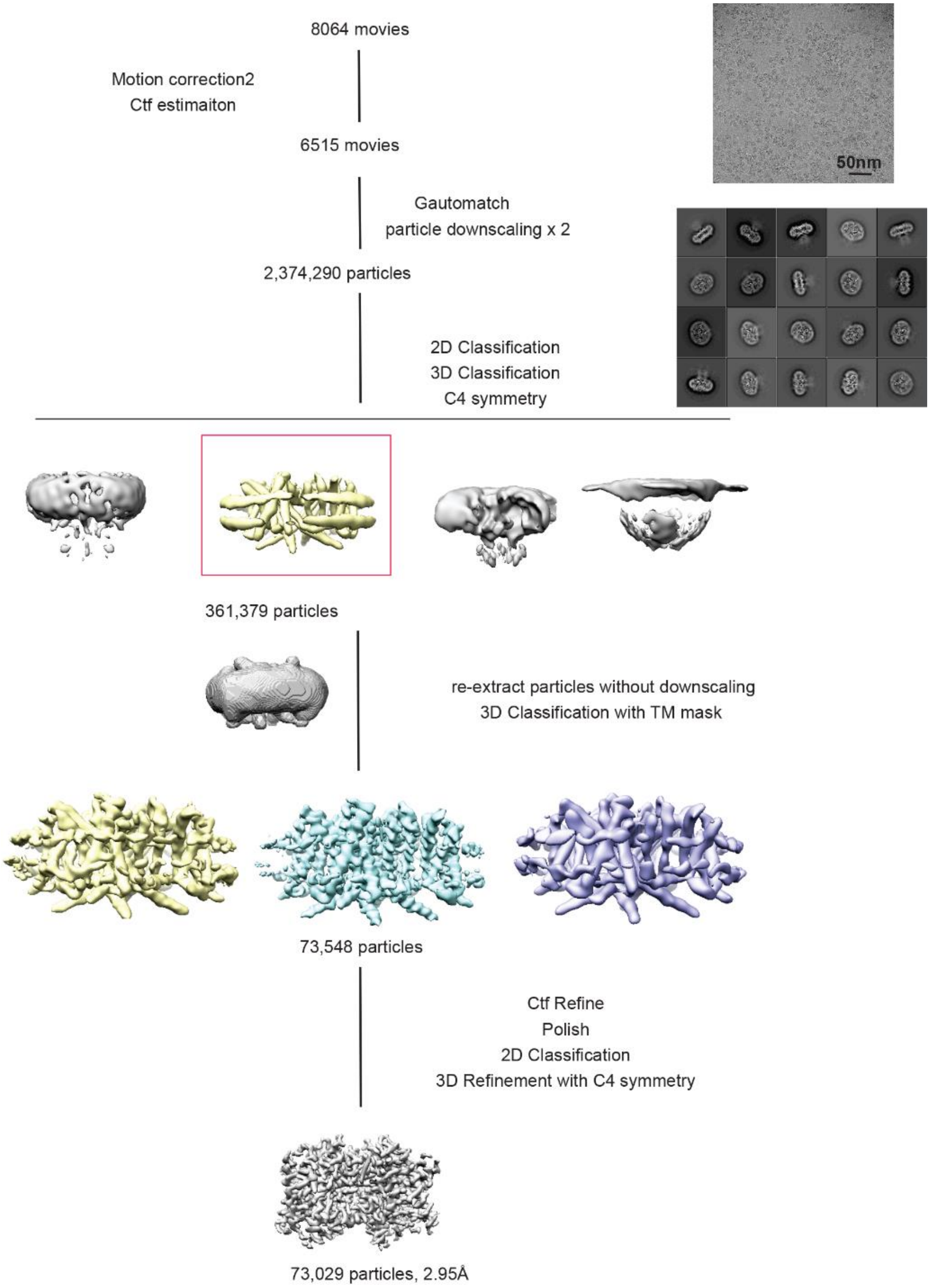

Supplementary Information Figure 1|

Data processing workflow for the structure of Kv2.1 (1-598)

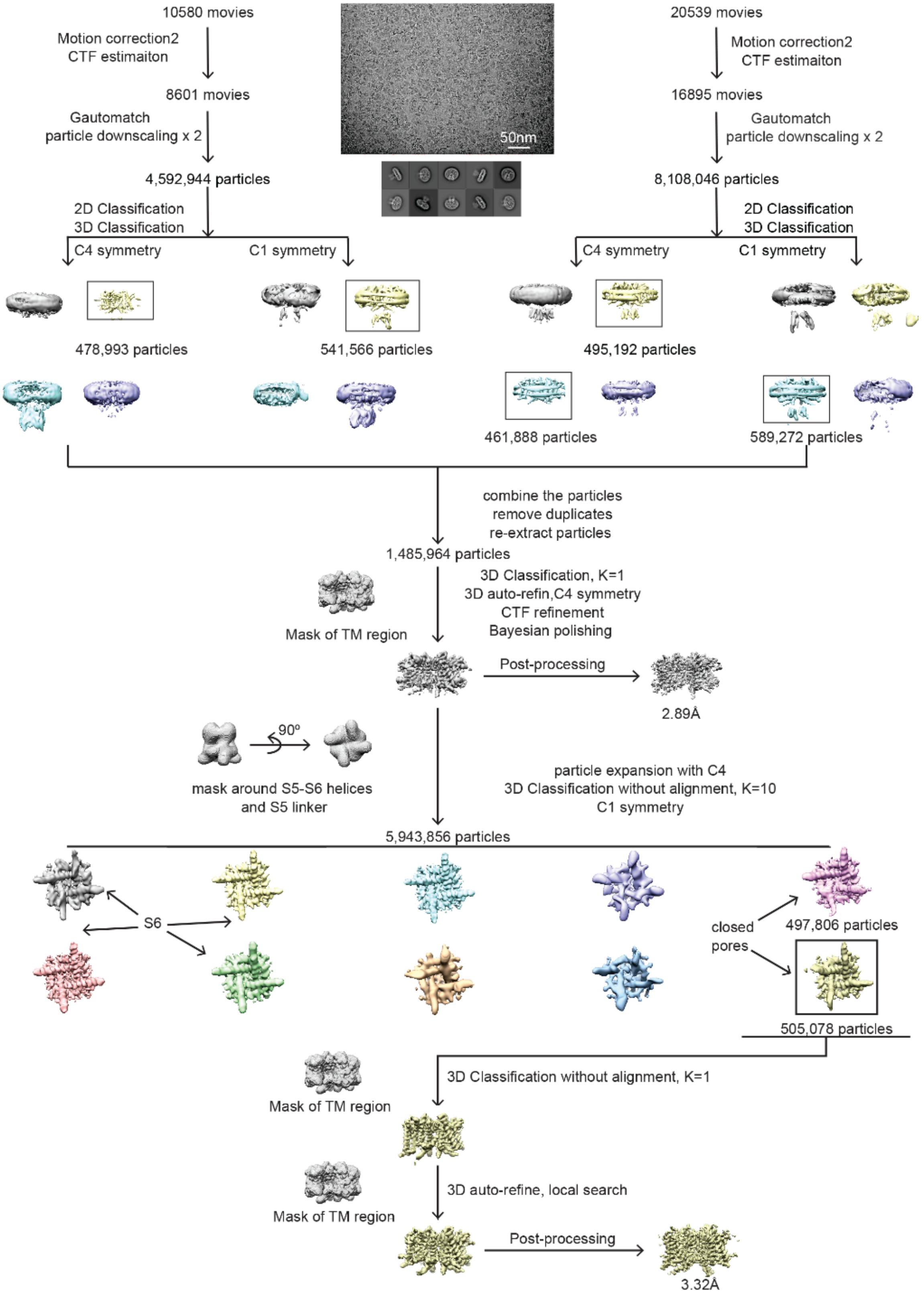

Supplementary Information Figure 2|

Data processing workflow for the structure of the L403A mutant of Kv2.1 (1-598)

Supplementary Information Table 1.
Gating properties of epileptic encephalopathy mutations in Kv2.1.

	$V_{1/2}$ (mV)	SEM	z	SEM	n	y
Kv2.1 WT	-4.8	2.0	3.7	0.2	4	2
S198F	51.1	3.3	1.1	0.1	5	2
T206K	13.7	4.5	1.0	0.1	3	2
T206M	11.1	4.6	1.7	0.3	3	2
L207P	-9.4	5.4	1.2	0.2	3	2
R302C	-82.0	3.5	1.6	0.4	5	2
R308H	71.6	3.3	0.9	0.0	6	4
K387N	-15.1	1.0	5.1	0.7	6	6

Voltage-activation (G-V) relations were obtained using tail current measurements in 100 mM external K^+. The Boltzmann equation was fit to G-V relations to obtain values of $V_{1/2}$ (midpoint) and z (steepness). Values of n indicate the number of cells studied and values of y indicate the number of independent experiments.

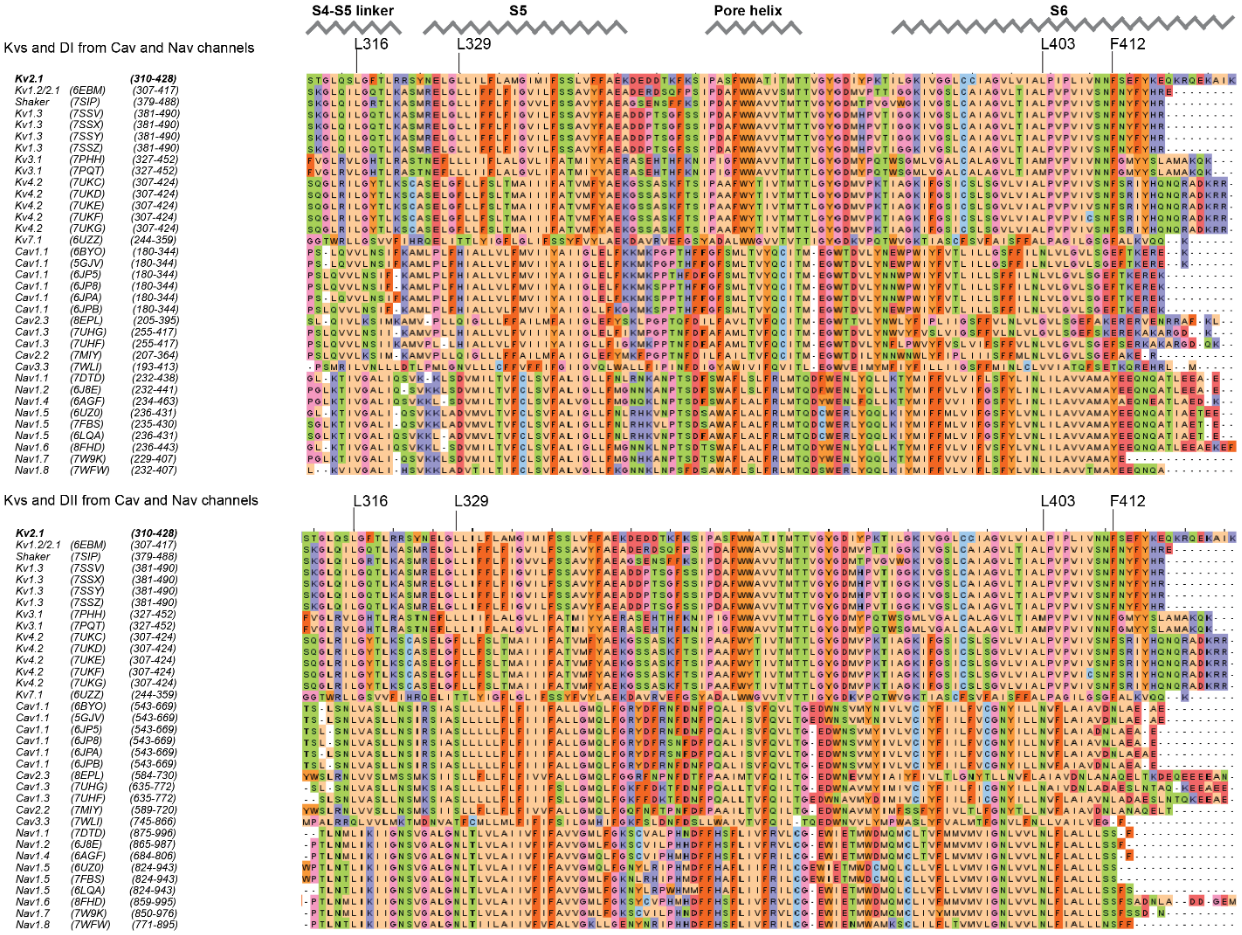
S4-S5 linker
S5
Pore helix
S6
Kvs and DI from Cav and Nav channels
L316
L329
L403
F412
Kvs and DII from Cav and Nav channels
L316
L329
L403
F412

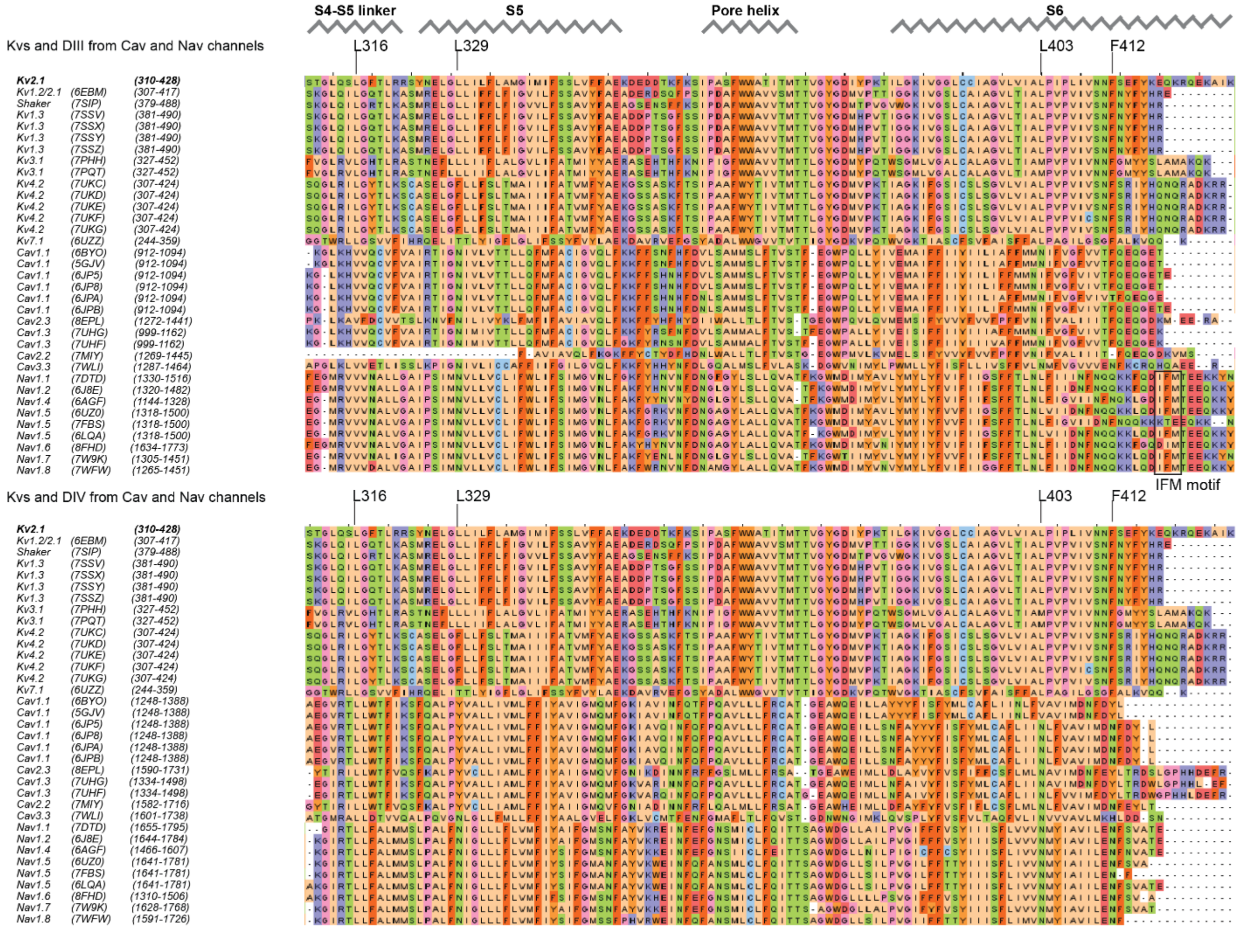

Supplementary Information Figure 3|
Structure-based sequence alignment of Kv, Cav and Nav channels

Structure-based sequence alignment of residues from the S4-S5 linker to the end of S6 with residues colored based on side chain character. Residues identified within the hydrophobic coupling nexus are highlighted as well as other regions of interest (IFM motif).

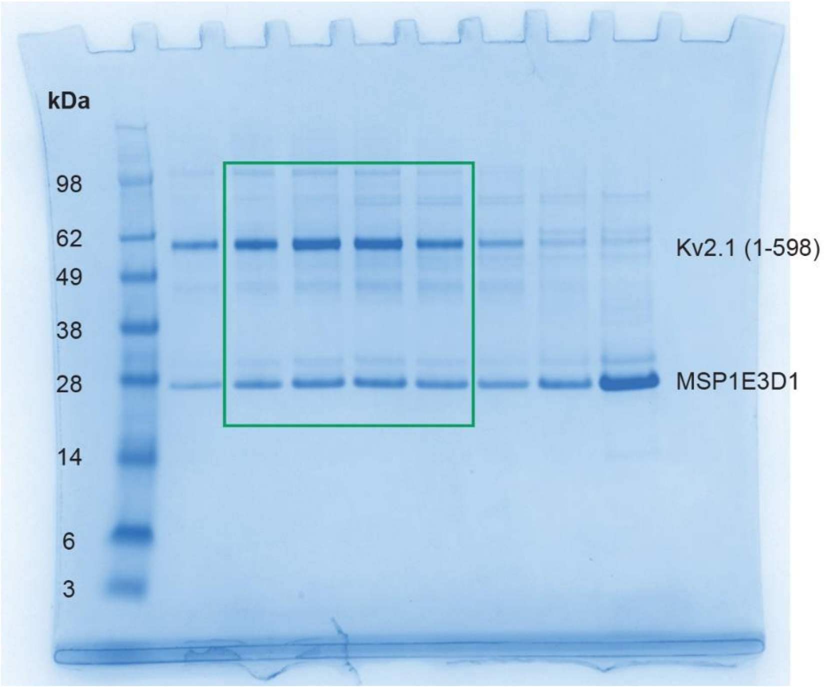

Supplementary Information Figure 4|

Uncropped gel from Extended Data Fig. 1d
